# Cell Adhesion Molecule 1 (CADM1) Is an Independent Prognostic Factor in Patients with Cutaneous Squamous Cell Carcinoma

**DOI:** 10.3390/diagnostics11050830

**Published:** 2021-05-04

**Authors:** Natsuko Saito-Sasaki, Yu Sawada, Etsuko Okada, Motonobu Nakamura

**Affiliations:** Department of Dermatology, University of Occupational and Environmental Health, Kitakyushu, Fukuoka 807-8555, Japan; e-okada@med.uoeh-u.ac.jp (E.O.); motonaka@med.uoeh-u.ac.jp (M.N.)

**Keywords:** CADM1, cutaneous squamous cell carcinoma, prognosis

## Abstract

Cell adhesion molecular 1 (CADM1) is a multifunctional cell adhesion molecule belonging to the immunoglobulin superfamily, which suppresses malignant solid tumor development. However, the correlation between CADM1 expression and prognosis in cutaneous squamous cell carcinoma (cSCC) patients remains unclear. In a retrospective analysis of 88 patients diagnosed with cSCC at our institution between January 2006 and December 2016, the degree of CADM1 expression in tumor cells was evaluated by immunostaining. Fifty-five and 33 patients had tumors with high and low CADM1 expression, respectively. Low CADM1 expression on the tumor was associated with poor differentiation, whereas the Kaplan–Meier curve and log-lank test indicated a favorable prognosis with high CADM1 expression. Multivariate analysis excluding the effect of the degree of differentiation and clinical stages showed that the hazard ratio (HR) of survival was significantly increased with low CADM1 expression. Thus, CADM1 expression is an independent prognostic factor for cSCC patients.

## 1. Introduction

Cutaneous squamous cell carcinoma (cSCC) is a major cutaneous malignancy derived from epidermal keratinocytes. The incidence rate of cSCC is increasing worldwide and has reached 15–35 per 100,000 individuals annually [1]. While the frequency of distant metastasis is relatively low, metastatic cSCC has a poorer prognosis, with a 25–35% five-year survival rate [2]. Therefore, it is essential to find a marker to predict the prognosis of cSCC. As risk factors of cSCC, UV radiation and human papilloma virus infection are known to be associated with the risk of SCC. Indeed, several key molecules in the mechanisms of the development of SCC have been identified, such as p53 and retinoblastoma protein (pRb) [3]. Ultraviolet radiation-induced mutations in the p53 tumor suppressor gene and human papilloma virus inhibition of the p53 and retinoblastoma tumor suppressor gene products appear to play significant roles in the development of cSCC [4]. Indeed, a previous study showed that 7,12-dimethylbenzanthrancene (DMBA) and 12-O-tetradecanoylphorbol-13-acetate (TPA) were frequently used as a cSCC initiator and promoter respectively in a mouse model [5], and p53-deficiency in epidermis contributes to the development of the tumor size of SCC by this DMBA/TPA treatment SCC model [6]. Therefore, a knowledge of cSCC risk factors is important for clinicians to evaluate the disease condition of cSCC patients.

In the first step of tumor metastasis, tumor cells need to decrease their expression of adhesion molecules to detach from the tumor nest. Among adhesion molecules, cell adhesion molecule 1 (CADM1) has recently been attracting attention as a candidate therapeutic target in various tumors. CADM1 belongs to the immunoglobulin superfamily, which is expressed in various normal tissues and organs including the nervous system, mast cells, testis, and lungs [7,8]. CADM1 plays an important role in suppressing malignant solid tumor cell invasion and metastasis [9]. CADM1 expression is inversely correlated with clinical stage progression, lymph node metastasis, and vascular invasion in lung cancer [10,11]. However, the actual role of CADM1 in cSCC remains unclear.

As a prognostic factor, tumor differentiation is already known to determine clinical prognosis. A poorly differentiated type of tumor shows decreased expression of adhesion molecules [12] and a high frequency of metastatic lesions. Chronic skin damages such as ultraviolet light exposure, burn scars, or arsenic exposure are known to act as triggers to provoke cSCC [13]. Chronic skin damage-triggered cSCC tends to show an unfavorable clinical behavior [14]. However, the relationship between CADM1 and these clinical factors remains unclear. Moreover, these clinical factors affect the prognosis of cSCC. Thus, the contribution of CADM1 to cSCC prognosis should also be analyzed considering the influence of these clinical factors.

To clarify these issues in this study, we examined the correlation between the CADM1 expression level in cSCC and its prognosis by statistical analysis.

## 2. Materials and Methods

### 2.1. Patient Population

In total, 88 patients who underwent surgery as an initial treatment for cSCC at the Department of Dermatology, University of Occupational and Environmental Health were enrolled in this study during the 10-year period between January 2006 and December 2016. Diagnosis was based on clinical and histopathological features, as described previously [15]. Tissue specimens were obtained from patients who underwent surgery at our institution. All the tumors were confirmed as cSCC by pathologists and were classified according to the tumor-node-metastasis (TNM) classification of the 2017 American Joint Committee on Cancer guidelines, 8th edition [16].

### 2.2. Clinical Evaluation

The patients were categorized according to the degree of CADM1 expression, age, sex, the presence of chronic sun damage, tumor differentiation, and the TNM stage. Chronic sun damage was defined by the localization of cSCC at a sun exposure site. The classification of tumor cell differentiation was performed by different pathologists [15].

### 2.3. Immunostaining for CADM1

Immunochemical staining for CADM1 was conducted using a CADM1 monoclonal antibody (mAb, Lifespan Bioscience, Inc., Seattle, WA, USA) on formalin-fixed, paraffin-embedded specimens [17]. In brief, specimens were cut into 4-μm-thick sections and then deparaffinized in xylene and dehydrated through graded alcohol solutions. Antigen retrieval was conducted with boiling in citrate buffer, pH 6.0, using a microwave treatment. All sections were treated with methanol containing 0.3% H_2_O_2_ for 15 min to block endogenous peroxidase activity. Immunoglobulin G was treated using normal rabbit serum (Nichirei, Tokyo, Japan) to avoid nonspecific antibody binding. After an overnight incubation at 4 °C with mouse anti-CADM1 mAb (Lifespan BioSciences, Inc., Seattle, WA, USA), the sections were incubated with biotinylated rabbit-anti-mouse secondary antibody (Nichirei, Tokyo, Japan) followed by incubation in a streptavidin-peroxidase complex solution for 30 min. Signals were generated by incubation with 3-amino-9-ethyl carbazole to visualize the immunostaining.

### 2.4. Analysis of CADM1 Expression Intensity

The specimens were digitized using the Nano Zoomer Digital Pathology C9600 system (Hamamatsu Photonics, Hamamatsu, Japan). Analysis of the expression intensity in histological specimens was performed using the following processes, as described previously [17,18,19,20]. The results were exported to JPG files and were opened in Adobe Photoshop CS(J) (Adobe Systems, Inc., San Jose, CA, USA). Five different areas from the tumor cell cytoplasm were selected and expressed as Red channel histograms. Histograms revealed 255 different shades from pitch black (0) to pure white (255), and a number represented the level of brightness for each color. We analyzed the mean intensity of the histogram in the cytoplasm and averaged the value of five different areas. To obtain the density, we calculated the 255 “mean” of each color. We called these “red density” (RD) values and used them for further investigation. Specimens with an RD value <90 were defined as the low expression group, and specimens with an RD value ≥90 were defined as the high expression group.

### 2.5. Statistical Analyses

Fisher’s exact test for unpaired data was used to analyze the association between CADM1 expression and various clinicopathological factors. Univariate and multivariate analyses of the overall survival were conducted with the log-rang test, and Kaplan–Meier curves were generated. The overall survival was calculated from the date of the first diagnosis to the date of death or the latest contact with the patient. Multivariate comparisons were made using the Cox proportional hazards model. Univariate and multivariate analyses were performed using EXR (Saitama Medical Center, Jichi Medical University, Saitama, Japan), a graphical user interface for R (The R Foundation for Statistical Computing, Vienna, Austria). Kaplan–Meier survival analyses, Student’s *t*-tests, one-way Analysis of variance (ANOVA), and Fisher’s test were performed using GraphPad Prism 4.0, a modified version of the R commander designed to add the statistical functions frequently used in biostatistics [21].

### 2.6. Quantitative Real-Time PCR

Total RNA was extracted from paraffin-embedded sections using NucleoSpin total RNA FFPE (Takara Bio Inc., Otsu, Japan) according to the manufacturer’s instructions, as previously described [22,23]. Complementary DNA (cDNA) was reverse-transcribed from total RNA samples using the PrimeScrip II 1st strand cDNA Synthesis Kit (Takara Bio Inc.). cDNA was used in quantitative real-time PCR using the TaqMan Universal PCR Master Mix (Thermo Fisher Scientific, Waltham, MA, USA) and TaqMan Gene Expression Assays (Thermo Fisher Scientific). Gene expression was measured on a Step One Plus Real-Time PCR System (Applied Biosystems, Foster, CA, USA) and was determined by the 2^−ΔΔCt^ method. The results were normalized to those of the housekeeping mRNA of glyceraldehyde-3-phosphate dehydrogenase (GAPDH). To analyze the difference in CADM1 gene expression, tumors were classified as metastatic and non-metastatic tumors based on pathological analysis. Total RNA was extracted from the paraffin-embedded samples of four patients with metastatic tumors and 21 patients with non-metastatic tumors, and these samples were further analyzed by quantitative real-time PCR analysis. The classification of non-metastatic and metastatic tumors was defined by the clinical tumor stages (T stage) I–II and III–IV, respectively.

### 2.7. Microarray Data Analysis

For the microarray data analysis, CADM1 mRNA expression data for 25 cSCC patients were obtained from a public data set deposited in the National Center for Biotechnology Information (NCBI) Gene Expression Omnibus (GEO) database (GEO accession no. GDS3292) [24].

### 2.8. Study Approval

Our retrospective, nonrandomized, observational study using existing data was reviewed and was approved by the Institutional Review Board at the University of Occupational and Environmental Health in accordance with the Declaration of Helsinki (Approval number: H30-035) (Approval date: 20 September 2018). All methods were carried out in accordance with relevant guidelines and regulations. Because this study was a retrospective cohort study, the opt-out method of obtaining informed consent was adopted in this study, and informed consent was waived by the Institutional Review Board at the University of Occupational and Environmental Health.

## 3. Results

### 3.1. Clinical Patient Profiles

The clinical data of 88 patients (ratio of male to female: 53:35) are summarized in Table 1. The median patient age was 81 years (range: 37–99 years). Forty-six patients had chronic sun damage. Fifty-nine patients had good differentiation, 20 patients had moderate differentiation, and nine patients had poor differentiation. According to the AJCC staging system, 19 patients (21.5%) had stage I, 58 patients (65.9%) had stage II, eight patients (9.1%) had stage III, and three patients (3.4%) had stage IV.

### 3.2. CADM1 Expression and Its Clinical Differences

**Figure 1:**
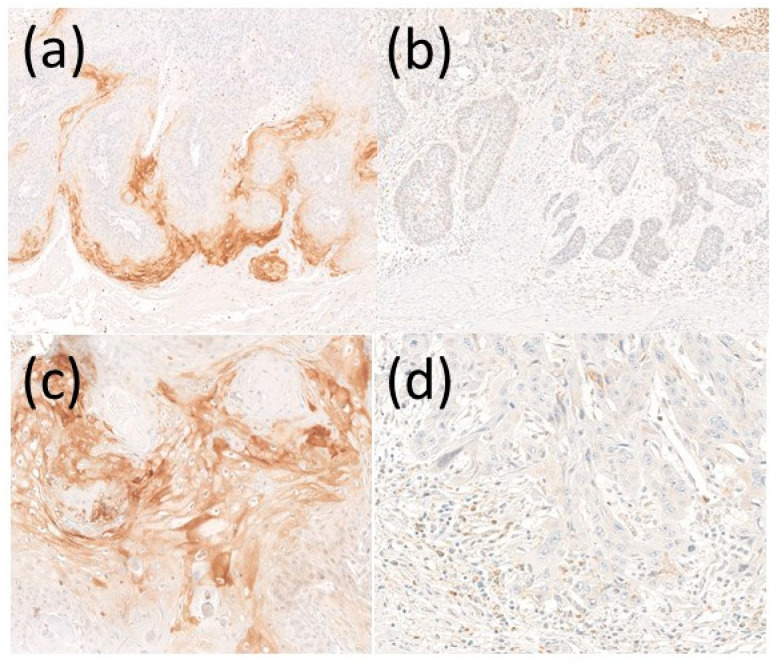
Representative CADM1 expression on cSCC. CADM1 expression was analyzed by immunohistochemical staining in patients with (**a**) high CADM1 expression and (**b**) low CADM1 expression. (**c**) High magnification view of high CADM1 and (**d**) low CADM1 expression.

Primary tumors from each patient were stained immunohistochemically with an anti-CADM1 antibody. Representative photos of high and low CADM1 expression are shown in Figure 1a,b, respectively. CADM1 was ordinally expressed in the invasive front of the tumor (Figure 1a). On the contrary, low expression of CADM1 cases exhibited a weak expression of CADM1 (Figure 1b). The positive CADM1 is mainly located in the marginal region of the tumor rather than in the center or randomly. Next, the intensity of CADM1 expression was analyzed. We classified CADM1 expression into two groups: RD values over 90 as the high CADM1 group, and RD values under 90 as the low CADM1 group (Figure 1c,d). There were 56 patients in the high CADM1 expression group and 32 patients in the low CADM1 expression group.

Next, we compared the clinicopathological variables between the CADM1 high and low expression groups (Table 2). Interestingly, low CADM1 expression was significantly more frequent in patients with poorly differentiated tumors (*p* < 0.01, Table 2). Because it has been reported that poor differentiation changes in cSCC are closely related with an unfavorable clinical behavior [15], we attempted to explore whether these clinical factors were correlated with patient survival.

### 3.3. Survival of cSCC Patients

**Figure 2:**
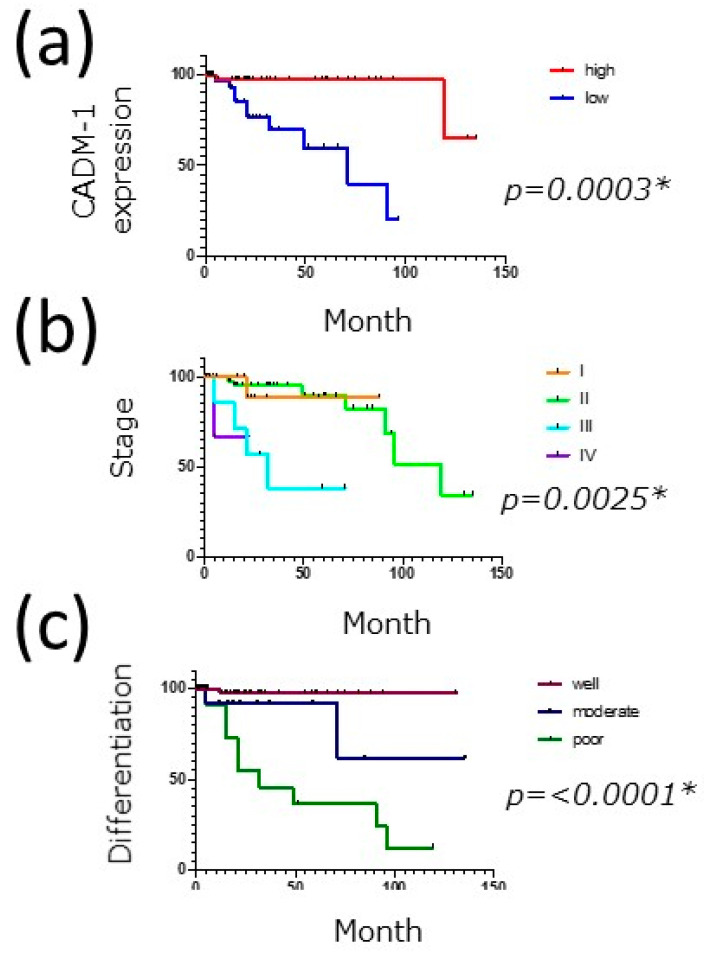
OS in patients with cSCC. Kaplan–Meier curves were generated by OS with differences in the (**a**) CADM1 expression, (**b**) clinical stages, and (**c**) differentiation. The *p*-value was evaluated by the log-rank test. * indicates a statistical significant difference.

First, to examine the impact of CADM1 on the survival of cSCC patients, the survival rates for the tumor were evaluated using the Kaplan–Meier method. The overall survival (OS) rate was significantly better in the high-expression group than in the low-expression group (Figure 2a) according to the Kaplan–Meier survival analyses and log-rank tests. These findings suggest that CADM1 expression might contribute to the survival of cSCC patients.

Furthermore, we also analyzed the contribution of clinical factors to the survival of cSCC patients. In addition, OS in cSCC was correlated with poor differentiation and significantly unfavorable behavior (Figure 2b), as shown in a previous study [25]. The survival curves of OS became significantly worse as the tumor stage advanced, and there was a significant difference between well-differentiated and poorly differentiated cSCC (Figure 2c). Therefore, these results suggest that these clinical factors might affect the difference in survival of cSCC patients in association with the CADM1 expression level.

### 3.4. Univariate and Multivariate Analysis for Survival

Finally, to confirm whether CADM1 is an independent prognostic factor, we performed univariate and multivariate analyses of each expression in comparison with the clinical factors shown in Table 3. In the univariate analysis, the CADM1 high-expression group showed a significantly better OS (Table 3). As shown in Figure 2, clinical factors such as differentiation and clinical stage (TNM Stage IV) also exhibited significant differences in OS and PFS (Table 3). Both clinical factors are important prognostic indicators for cSCC, and our study also confirmed their importance in OS in patients with cSCC. Since differentiation is associated with the degree of cell adhesion molecules in cSCC [26], we next investigated whether CADM1 was independently related to their prognosis. To exclude the possible influence of these factors in the CADM1-related prognostic significance, a multivariate analysis was conducted. In addition to the clinical stage (TNM stages III and IV), the multivariate analysis identified that the HR of low CADM1 expression was significantly higher than that of the high expression group (Table 3). These findings demonstrate that CADM1 expression is an independent prognosis factor of cSCC.

### 3.5. Decreased CADM1 Expression in Invading cSCC Tumors

**Figure 3:**
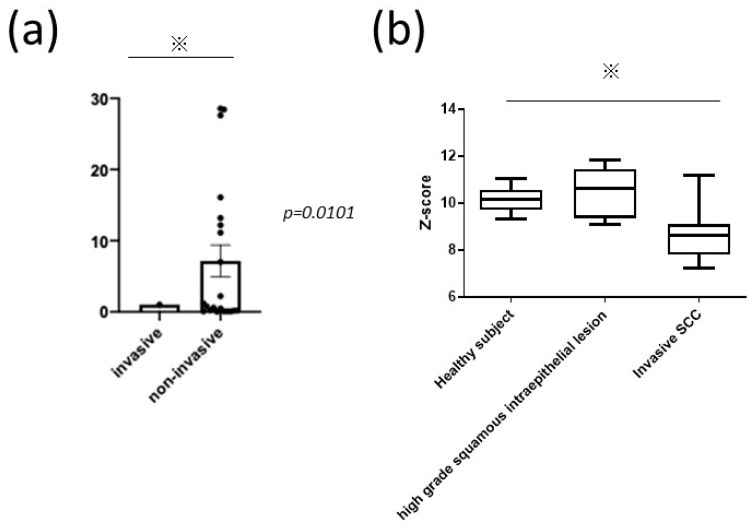
Gene expression of CADM1 and microarray dataset analysis. (**a**) Difference in CADM1 gene expression between non-metastatic and metastatic cSCC patients. (**b**) Microarray analysis using a previously published dataset. The *p*-value was evaluated by a Student’s *t*-test and one-way ANOVA. * indicates a statistical significant difference.

Finally, we examined the mRNA expression of CADM1 in human cSCC lesions from our patients and verified our results using a public microarray dataset. RT-PCR experiments showed that CADM1 expression was significantly lower in metastatic cSCC specimens than in non-metastatic specimens (Figure 3a). An analysis of the microarray dataset revealed that CADM1 mRNA levels were significantly decreased in patients with invasive cervical SCC compared with those in healthy subjects and patients with noninvaded cervical SCC (Figure 3b), which was the different origin of SCC. However, this result might support the finding that CADM1 expression is related to the progression of SCC, without limiting oneself to cervical SCC but also including cSCC. These results suggested that CADM1 may negatively regulate tumor invasion. Consistent with this, there were 21 patients with lymphovascular invasion in our study, and the CADM1 high and low groups included eight and 13 patients, respectively, with a significant difference in the frequency of lymphovascular invasion between the CADM1 high and low groups (*p* = 0.0086). Although our study could not conduct an in vitro study to confirm the actual impact of the migration of tumor cells, these results suggest that CADM1 might be a key molecule for predicting tumor prognosis in cSCC.

## 4. Discussion

Recent studies identified that CADM1 was closely related to the development or regulation of skin cancers [9], such as SCC [25], malignant melanoma [27], adult T-cell leukemia/lymphoma [28], mycosis fungoides [17,29], Sézary Syndrome [30], and Merkel cell carcinoma [31].

In this study, we examined the localization of CADM1 expression in cSCC tumors and identified that the degree of CADM1 expression was correlated with patient survival. A multivariate analysis revealed that CADM1 was an independent prognostic factor for cSCC patients. Thus, CADM1 might be one of the regulators in the development of cSCC. Our study may thus lead to a novel application of CADM1 in targeted therapy for cSCC.

Several studies support the impact of CADM1 on cSCC. Patients with decreased CADM1 expression were reported to demonstrate poor survival rates [25]. TSLC1 is related to the suppression of tumor proliferation and invasion, the cell cycle arrest at the G0/G1 phase, and the caspase-3-mediated induction of cell apoptosis [25]. Although this is a well-investigated study, it is difficult to exclude the influence of tumor differentiation and the clinical stage on their prognosis. Therefore, our study excluded their influence to prove the impact of CADM1 on cSCC prognosis.

In addition, a genome-wide study also suggested that CADM1 played a role in tumor development [32]. Consistently, our analysis using a public microarray dataset also showed that CADM1 mRNA levels were significantly decreased in patients with metastatic cSCC. Therefore, these results support the results of our study, indicating that CADM1 might be a key molecule in cSCC development.

CADM1 was expressed in the marginal region of the tumor, whereas loss of CADM1 expression was observed in unfavorable clinical cases. These differences in CADM1 strength are reasonable in accordance with the tumor expansion mechanisms, because low CADM1 expression facilitates tumor cell detachment from tumor nests and might cause invasion and distant metastasis. Although this study could not elucidate a more detailed molecular mechanism of the impact of CADM1 on tumor metastasis and invasion, it might be possible to predict tumor prognosis by examining the pattern of CADM1 expression in the tumor.

Contrarily to solid tumors, nonsolid tumors such as mycosis fungoides and adult T-cell leukemia/lymphoma [33,34,35,36] showed that high expression of CADM1 had an unfavorable clinical behavior [17,29,37]. In nonsolid tumors, CADM1 contributes to the invasion of the tumor into vessels and other organs, suggesting that the prognostic significance of CADM1 is different in the types of tumors.

Differentiation of malignant tumors is one of the determinant factors for their prognosis. Some reports have shown that E-cadherin and other adhesion molecules tend to decrease in poorly differentiated types of tumors [26]. Indeed, CADM1 expression was decreased in the poorly differentiated type specimens in our study. Moreover, CADM1 expression has a significant relationship with tumor differentiation. Therefore, it is important to distinguish the direct impact of CADM1 on tumor prognosis by excluding the effect of tumor differentiation. Because our study identified CADM1 as one of the independent prognostic indicators by a multivariate analysis, we suggest that CADM1 targeted therapy may be beneficial for tumor treatment.

Chronic skin damage is known as a trigger that causes cSCC and also affects its prognosis. Chronic skin damage-related precancerous lesions show decreased expression of cell adhesion molecules [38]. On the contrary, some studies have reported that precancerous lesions exhibit normal or increased expression of E-cadherin [39]. Therefore, the influence of chronic skin damage on the expression of adhesion molecules in cSCC is currently controversial. Because cSCC arising from scars shows a high frequency of local recurrence and metastasis [14,38], we considered that chronic skin damage might also be associated with CADM1 expression. However, our study could not identify the prognostic significance of chronic skin damage and its relationship with CADM1 expression. Therefore, our results indicate that CADM1 expression might not be affected by previous chronic skin damage. Further experiments are needed to clarify these issues by analyzing a larger number of patients with chronic skin damage.

Our study also showed that the clinical stage was an independent prognostic factor of cSCC by multivariate analysis. CADM1 expression reflects a tumor-side factor, whereas the clinical stage is influenced by both the tumor and host factors. As a host-side factor, tumor immunity in host defense is one of the critical factors for determining patient prognosis [39]. The metastatic cascade is a complex multistep process, and the role of CADM1 expression is not restricted to cell–cell adhesion. However, it might also affect the infiltration of inflammatory cell migration into the tumor [9]. Indeed, CADM1 has a potent threshold for triggering the immune responses of immune cells such as NK-cell and CD8+ T-cell [40]. These findings suggest that loss of CADM1 might also contribute to the escape phenomenon against antitumor immunity due to a decreased threshold for immune cells to infiltrate into the tumor. Therefore, CADM1-targeted treatment might also have a potential to be used in combination with immune check point treatments in the future.

Taken together, our study showed CADM1 as an independent prognostic factor in cSCC. Although our study could not clarify the molecular-based mechanism of the CADM1-related development of cSCC, therapeutic applications for CADM1 might be desired for the detailed regulatory mechanisms of CADM1 in the tumor.

## Figures and Tables

**Table 1:** Clinical characteristics.

Total Number	88
CADM I High Low	56 32
Age (years) <75 ≥75	25 63
Sex Male Female	53 35
Chronic sun damage Negative Positive	42 46
Differentiation Good differentiation Moderate differentiation Poor differentiation	59 20 9
TNM stage I II III VI	19 58 8 3

**Table 2:** Relationship between the CADM1 expression and clinicopathological variables of CSCC.

Variable	CADM1 Expression		*p* Value
	High (*n* = 53)	Low (*n* = 35)	
Age (years) <75 ≥75	14 (26.4%) 39 (73.6%)	11 (31.4%) 24 (68.6%)	0.636
Sex Male Female	30 (56.6%) 23 (43.4%)	23 (65.7%) 12 (34.3%)	0.505
Chronic sun damage Negative Positive	21 (39.6%) 32 (60.4%)	21 (60.0%) 14 (40.0%)	0.082
Differentiation Good differentiation Moderate differentiation Poor differentiation	42 (79.2%) 10 (18.2%) 1 (1.8%)	17 (48.6%) 10 (28.6%) 8 (22.9%)	<0.01
TNM stage I II III VI	15 (28.3%) 34 (64.2%) 2 (3.8%) 2 (3.8%)	4 (11.4%) 24 (68.6%) 6 (17.1%) 1 (2.9%)	0.060

*p*-value was evaluated by Fisher’s test.

**Table 3:** Univariate and multivariate analyses.

Variable	Univariate OS		Multivariate OS	
	HR (95% CI)	*p*	HR (95% CI)	*p*
CADM1 expression High Low	1 12.95 (2.93–57.22)	<0.001	1 1 7.83 (1.82–33.65)	<0.01
Age (years) <75 ≥75	1 1.17 (0.43–3.16)	0.747	1 0.64 (0.15–2.57)	0.529
Sex Male Female	1 0.55 (0.19–1.56)	0.265	1 0.35 (0.09–1.41)	0.141
Chronic sun damage Negative Positive	1 0.71 (0.28–1.83)	0.481	1 2.01 (0.70–5.733)	0.191
Differentiation Good Moderate Poor	1 2.28 (0.63–8.05) 6.82 (2.30–20.17)	0.209 < 0.001	1 1.35 (0.36–4.99) 1.48 (0.34–6.44)	0.65 0.60
TNM stage I II III VI	1 1.75 (0.21–14.14) 6.60 (0.74–59.2) 19.27 (1.69–219.5)	0.599 0.091 <0.05	1 2.29 (0.24–20.79) 42.28 (3.49–511.8) 18.28 (1.06–315.1)	0.464 <0.05 <0.05

## Data Availability

All data that support the findings of this study are included in this manuscript.
